# Análise da Repolarização Ventricular em Pacientes Hipertensos: Influência do Fenômeno de Redução da Pressão Arterial durante a Noite

**DOI:** 10.36660/abc.20240725

**Published:** 2025-04-29

**Authors:** Marco Aurélio Goulart, Dalmo Antonio Ribeiro Moreira, Fernando Yue Cesena, Jonathan Batista Souza, Antonio Gabriele Laurinavicius, Fernanda Marciano Consolim-Colombo, Márcio Gonçalves de Sousa

**Affiliations:** 1 Instituto Dante Pazzanese de Cardiologia São Paulo SP Brasil Instituto Dante Pazzanese de Cardiologia, São Paulo, SP – Brasil; 2 Hospital das Clínicas Faculdade de Medicina Universidade de São Paulo São Paulo SP Brasil Instituto do Coração do Hospital das Clínicas da Faculdade de Medicina da Universidade de São Paulo, São Paulo, SP – Brasil

**Keywords:** Hipertensão, Fatores de Risco de Doenças Cardíacas, Eletrofisiologia Cardíaca

## Abstract

Alterações na repolarização ventricular estão associadas a arritmias ventriculares e maior mortalidade. A associação entre o padrão pressórico não dipper e as alterações na repolarização ventricular continua sendo controversa. O objetivo principal é comparar as medições da repolarização ventricular (intervalo QT, QTc, Tp-Tf, Tp-Tf/QT e QTd) em hipertensos dippers e não dippers. Os objetivos secundários são comparar as medições entre pacientes hipertensos controlados e não controlados, assim como entre hipertensos resistentes e não resistentes. Este estudo observacional e transversal envolveu pacientes monitorados em um Serviço de hipertensão. O nível de significância adotado para a análise estatística foi 5%. Foi admitido um total de 130 participantes. A média de idade foi de 67,4 anos, com 72% apresentando alguma forma de lesão de órgãos-alvo. As medições de repolarização não apresentaram diferenças entre dippers e não dippers. No entanto, dentro do grupo de hipertensão resistente em comparação com o grupo não resistente, foram observadas diferenças no intervalo QT em V5 (433,3 ms vs. 420,9 ms, p = 0,046), Tp-Tf em V2 (85,4 ms vs. 78,7 ms, p = 0,049) e em V5 (84,6 ms vs. 74,6 ms, p = 0,006), Tp-Tf/QT em V5 (0,19 vs. 0,18, p = 0,019), índice de Sokolow-Lyon (18,8 mm vs. 15,7 mm, p = 0,011) e índice de Cornell (14,2 mm vs. 11,2 mm, p = 0,002), com valores ajustados pela idade. Nessa população hipertensa de alto risco cardiovascular, não foi encontrada diferença nas medições de repolarização entre dippers e não dippers. Entretanto, este é o primeiro estudo a demonstrar o aumento das medições de repolarização ventricular em pacientes com hipertensão resistente.

## Graphical abstract

**Figura Central:**
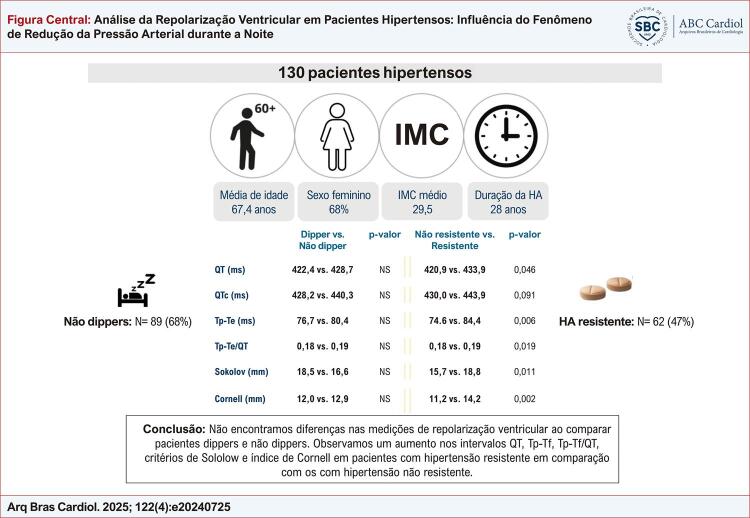
Análise da Repolarização Ventricular em Pacientes Hipertensos: Influência do Fenômeno de Redução da Pressão Arterial durante a Noite

**Figura Central:** Análise da Repolarização Ventricular em Pacientes Hipertensos: Influência do Fenômeno de Redução da Pressão Arterial durante a Noite

Análise da repolarização ventricular em pacientes hipertensos: influência do fenômeno de redução da pressão arterial durante a noite.

## Introdução

As alterações na repolarização ventricular desempenham um papel importante na formação de arritmias ventriculares e estão associadas a maior mortalidade. Os intervalos QT e QTc, especialmente medidos em DII e V5, representam um resultado da repolarização ventricular.^1-3^

A dispersão do QT (QTd), que é a diferença entre o intervalo QT mais longo e o mais curto entre as 12 derivações, visa estimar a diferença de tempo da repolarização ventricular nas diversas regiões do coração.^4,5^ O intervalo Tp-Tf (Tpico a Tfinal) representa a repolarização transmural, envolvendo o epicárdio, o endocárdio e o mesocárdio.^6-9^

Em pacientes com hipertensão arterial (HA), as alterações na repolarização ventricular decorrem da remodelação elétrica causada pela hipertrofia dos cardiomiócitos, depósito de colágeno e isquemia miocárdica devido ao desequilíbrio entre oferta e demanda.^10,11^ No entanto, em pacientes com padrão pressórico não dipper, a associação com as alterações na repolarização ventricular continua sendo controversa.^12-14^

Este estudo tem como objetivo comparar cinco medidas distintas de repolarização ventricular (intervalo QT, QTc, Tp-Tf, Tp-Tf/QT e QTd) ao comparar grupos de pacientes hipertensos dippers e não dippers. Os objetivos secundários incluem comparar as medições de repolarização ventricular ao dividir os entre pacientes hipertensos controlados e não controlados, assim como entre pacientes hipertensos resistentes e não resistentes.

## Métodos

### Desenho e população do estudo

Este é um estudo observacional e transversal envolvendo pacientes hipertensos monitorados no Departamento de Hipertensão de um hospital terciário, que foram submetidos ao monitoramento ambulatorial da pressão arterial (MAPA). Pacientes dippers e não dippers foram selecionados por meio desse exame.

Com base em um desvio padrão de 11,2 ms para a variável Tp-Tf identificada no estudo de Demir e Uyan,^12^ estimou-se um número de 130 pacientes para detectar diferenças de até 8 ms entre os grupos, com 90% de poder e um nível de significância de 5%.

Os critérios de exclusão incluíram pacientes que realizaram MAPA com menos de 70% de medições válidas, HA estágio 3 (definida por pressão arterial sistólica (PAS) ≥180 mmHg ou pressão arterial diastólica (PAD) ≥110 mmHg), cardiomiopatias (hipertrófica, isquêmica, congênita ou valvular com disfunção moderada ou importante), disfunção ventricular esquerda abaixo de 50%, doença arterial coronária obstrutiva diagnosticada, portadores de marca-passo, pacientes em hemodiálise, doença hepática grave, gravidez, HA secundária, neoplasia ativa em tratamento, IMC > 34,9, fibrilação atrial ou flutter, uso de amiodarona, propafenona, sotalol, verapamil ou diltiazem, eletrocardiograma não interpretável por outras razões e indivíduos já participantes de outras pesquisas.

### Coleta de dados

O exame de MAPA foi realizado utilizando o dispositivo DYNA-MAPA NG, com medições a cada 20 minutos durante o dia e a cada 30 minutos durante a noite. O período de sono foi baseado no relatório de eventos preenchido pelo paciente.

Pacientes com uma redução inferior a 10% na PAS e/ou PAD durante o sono em comparação com o período de vigília foram classificados como não dippers. A HA não controlada foi definida como PAS ≥130 mmHg e/ou PAD ≥80 mmHg com base no MAPA.

Os pacientes foram considerados portadores de hipertensão resistente se: 1) o MAPA revelou PAS ≥130 mmHg e/ou PAD ≥80 mmHg, apesar da terapia otimizada com um bloqueador dos canais de cálcio (BCC), diurético tiazídico e um bloqueador dos receptores da angiotensina (BRA) ou inibidor da enzima conversora da angiotensina (IECA); ou 2) o MAPA revelou PAS <130 mmHg e PAD <80 mmHg com o uso de doses otimizadas de BCC, diurético tiazídico e um BRA ou IECA, combinados com uma quarta classe de agentes anti-hipertensivos.

O eletrocardiograma de 12 derivações foi realizado utilizando o dispositivo Eletro System PRE0900001, padronizado com uma velocidade do papel de 25 mm/s e amplitude de 10 mm/mV. As medições foram realizadas pelo pesquisador com assistência semiautomática do software Eletro System V6.4.0.11.

Os intervalos QT e QTc foram medidos em todas as 12 derivações, mas apenas V5 foi usado como representação. O intervalo Tp-Tf foi medido em seis derivações no plano horizontal. As medições foram realizadas usando o método da tangente, em que uma linha tangente foi traçada na inclinação mais acentuada do último segmento da onda T até cruzar com a linha de base.

A correção do intervalo QT para frequência cardíaca foi realizada pela fórmula de Bazzett, aplicando-se o intervalo TR entre o complexo medido e o anterior. O índice de Sokolow-Lyon foi medido pela adição da amplitude da onda S em V1 à maior amplitude da onda R encontrada em V5 ou V6. O critério de Cornell foi estabelecido pela adição da amplitude da onda R na derivação aVL à amplitude da onda S na derivação V3.

O questionário da Escala de Sonolência de Epworth (ESE) e as medições da circunferência do pescoço foram coletados pelo investigador.

### Análise estatística

Variáveis contínuas foram expressas como médias ± desvios padrão (DP) ou medianas e faixas interquartil, conforme apropriado. As variáveis categóricas foram apresentadas como frequências absolutas e relativas. As comparações entre os grupos foram realizadas utilizando o teste t de Student não pareado (para variáveis contínuas com distribuição normal), o teste U de Mann-Whitney (para variáveis contínuas com distribuição não normal) ou o teste de Fisher (para variáveis categóricas). A normalidade da distribuição dos dados foi avaliada pelo teste de Anderson-Darling, juntamente com a inspeção visual de histogramas e gráficos quantil-quantil (gráficos Q-Q).

A associação do status de dipper ou não dipper às variáveis eletrocardiográficas foi avaliada por meio de modelos de regressão linear ajustados para a idade. Nesses modelos, a variável dependente foi a característica eletrocardiográfica (por exemplo, frequência cardíaca), a variável independente foi o fenótipo não dipper/dipper, e a idade foi considerada uma covariável. As estimativas, os intervalos de confiança de 95% e os p-valores, são apresentados correspondendo às diferenças ajustadas por idade nas variáveis dependentes entre os grupos não dipper e dipper. O mesmo procedimento foi realizado para estimar as diferenças ajustadas por idade nas medições eletrocardiográficas entre participantes com hipertensão controlada versus não controlada, assim como entre indivíduos com ou sem hipertensão resistente.

O nível de significância adotado para a análise estatística foi 5%. As análises estatísticas foram realizadas utilizando o software R (versão 4.1.2) e o software *jamovi* (versão 2.3.26.0). Esta pesquisa seguiu os princípios da Declaração de Helsinque e do Código de Nuremberg, em conformidade com as Normas de Pesquisa envolvendo Seres Humanos do Conselho Nacional de Saúde do Brasil (Res. CNS 196/96). Foi aprovada pelo Comitê de Ética em Pesquisa sob o parecer número 6.213.951, e todos os pacientes ou seus representantes legais assinaram o termo de consentimento livre e esclarecido.

## Resultados

Um total de 130 participantes foi admitido no estudo, com um tempo mediano de 3,8 semanas entre os exames de EKG e MAPA. A Tabela 1 mostra a proporção de pacientes classificados como dippers e não dippers com base nos resultados do MAPA.

**Tabela 1:** – Valores de pressão arterial obtidos pelo MAPA comparando os grupos de dippers e não dippers

Variáveis*	Dipper, N = 41	Não dipper, N = 89	p-valor
**PAS 24h (mmHg)**	124,3 (12,2)	122,8 (13,7)	0,534^†^
**PAD 24h (mmHg)**	75,6 (10,3)	73,6 (9,7)	0,291^†^
**PAS acordado (mmHg)**	128,6 (12,4)	123,7 (13,4)	0,042^†^
**PAD acordado (mmHg)**	79,2 (10,4)	75,1 (10,1)	0,035^†^
**PAS dormindo (mmHg)**	111,7 (11,5)	120,4 (16,2)	<0,001^†^
**PAD dormindo (mmHg)**	64,2 (9,8)	69,5 (9,9)	0,005^†^
**Redução anormal de PAS (%)**	12 (11, 14)	4 (-1, 7)	<0,001^§^
**Redução anormal de PAD (%)**	19 (15, 22)	7 (-1, 10)	<0,001^§^
**HA não controlada (%)**	19 (49%)	31 (35%)	0,151^‡^

^*^ valores expressos como média ± desvio padrão, mediana (25º percentil, 75º percentil) ou n (%). ^†^ p-valor calculado pelo teste t de Student. ^‡^ p-valor calculado pelo teste de Fisher. ^§^ p-valor calculado pelo teste U de Mann-Whitney. HA: hipertensão arterial; PAS: pressão arterial sistólica; PAD: pressão arterial diastólica.

A Tabela 2 mostra uma tendência de maior idade no grupo dos não dippers, embora sem significância estatística. Outras características também não apresentaram diferenças estatisticamente significativas.

**Tabela 2:** – Características demográficas, antropométricas e clínicas em pacientes hipertensos, divididos nos grupos dippers e não dippers

Variáveis*	Dipper, N = 41	Não dipper, N = 89	p-valor
**Idade (anos)**	66 (59, 73)	71 (64, 74)	0,095^§^
**Sexo feminino (%)**	27 (66%)	62 (70%)	0,688^‡^
**Raça (n/%)**			0,418^‡^
Brancos	23 (56%)	60 (67%)	
Pardos	6 (15%)	12 (13%)	
Negros	12 (29%)	17 (19%)	
**IMC**	30,0 (5,0)	29,3 (4,9)	0,476^†^
**Duração da HA (anos)**	26,0 (16,5, 38,5)	28,0 (22,0, 39,2)	0,348^§^
**ES Epworth (pts)**	7 (4, 15)	5 (2, 12)	0,289^§^
**Circunferência do pescoço (cm)**	36 (35, 39)	37 (35, 40)	0,419^§^
Homens	40,7	41,4	
Mulheres	34,8	35,8	

* valores expressos como média ± desvio padrão, mediana (25º percentil, 75º percentil) ou n (%). ^†^ p-valor calculado pelo teste t de Student. ^‡^ p-valor calculado pelo teste de Fisher. ^§^ p-valor calculado pelo teste U de Mann-Whitney. HA: hipertensão arterial; IMC: índice de massa corporal; ES: escala de sono.

A Tabela 3 demonstra um perfil de alto risco cardiovascular, com dislipidemia e pré-diabetes/diabetes presentes na maioria dos pacientes, assim como pelo menos uma lesão em órgão-alvo causada pela HA, sem diferença significativa entre os grupos.

**Tabela 3:** – Distribuição de comorbidades em pacientes hipertensos, divididos nos grupos dippers e não dippers

Variáveis*	Dipper, N = 41	Não dipper, N = 89	p-valor^**†**^
**Diabetes/ Pré-diabetes**	34 (83%)	80 (90%)	0,265
**Acidente vascular**	5 (12%)	14 (16%)	0,790
**Dislipidemia**	38 (93%)	86 (97%)	0,379
**Fumante/ Ex-fumante**	18 (44%)	28 (31%)	0,174
**Doença renal crônica**	11 (27%)	28 (31%)	0,683
**Lesão de Órgão-Alvo**	26 (63%)	67 (75%)	0,210
HVE	19 (46%)	41 (46%)	>0,999
Microalbuminúria	12 (29%)	30 (34%)	0,689
Retinopatia hipertensiva	13/32 (41%)	38/81 (47%)	0,675

* valores expressos como n (%). ^†^ p-valor calculado pelo teste Fisher. HVE: hipertrofia do ventrículo esquerdo.

O número médio de classes de medicamentos anti-hipertensivos utilizados foi três, sem diferença entre os grupos, exceto por uma maior prevalência do uso de simpatolíticos centrais (representados pela metildopa) no grupo dos dippers (Tabela 4).

**Tabela 4:** – Distribuição de medicamentos dividida nos grupos dippers e não dippers

Variáveis*	Dipper, N = 41	Não dipper, N = 89	p-valor
Nº de anti-hipertensivos	4 (3, 5)	3 (3, 4)	0,427^†^
IECA/BRA	39 (95%)	84 (94%)	>0,999^‡^
BCC	34 (83%)	68 (76%)	0,494^‡^
Diuréticos tiazídicos	33 (80%)	65 (73%)	0,391^‡^
Diuréticos de alça	1 (2,4%)	7 (7,9%)	0,434^‡^
Antagonista da aldosterona	16 (39%)	25 (28%)	0,229^‡^
Clonidina	2 (4,9%)	7 (7,9%)	0,719^‡^
Hidralazina	3 (7,3%)	5 (5,6%)	0,707^‡^
Nitrato	2 (4,9%)	2 (2,2%)	0,590^‡^
Doxazosina	1 (2,4%)	2 (2,2%)	>0,999^‡^
Betabloqueadores	20 (49%)	46 (52%)	0,851^‡^
Metildopa	6 (15%)	1 (1,1%)	0,004^‡^

* valores expressos como mediana (25º percentil, 75º percentil) ou n (%). ^†^ p-valor calculado pelo teste U de Mann-Whitney. ^‡^ p-valor calculado pelo teste de Fisher. IECA: enzima conversora da angiotensina inibidores; BRA: bloqueadores de receptor de angiotensina; BCC: bloqueadores de canal de cálcio.

Não houve diferenças nas medições ecocardiográficas entre os grupos. No entanto, vale ressaltar que o valor mediano do índice de volume atrial esquerdo (IVAE ml/m^2^) estava acima do valor de referência de 34 ml/m^2^ em ambos os grupos (Tabela 5).

**Tabela 5:** – Medições ecocardiográficas em pacientes hipertensos, divididos nos grupos dippers e não dippers

Variáveis*	Dipper, N = 41	Não dipper, N = 89	p-valor
**Diâmetro do AE (mm)**	39,2 (4,5)	39,2 (5,0)	0,951^†^
**IVAE (ml/m^2^)**	35 (30, 38)	35 (32, 40)	0,370^‡^
**LVEDD (mm)**	48 (45, 53)	47 (43, 51)	0,235^‡^
**LVESD (mm)**	31 (28, 33)	31 (28, 33)	0,691^‡^
**Diâmetro do SIV (mm)**	10 (9, 11)	10 (9, 10)	0,134^‡^
**Diâmetro do PP (mm)**	9 (9, 10)	9 (9, 10)	0,548^‡^
**Massa do VE (g)**	170 (134, 194)	150 (128, 186)	0,171^‡^
**Índice de massa do VE (g/m^2^)**	90,7 (22,9)	89,5 (20,2)	0,775^†^
**ERP**	0,38 (0,35, 0,42)	0,38 (0,35, 0,42)	0,765^‡^
**Disfunção diastólica**			0,206^§^
Não há	21 (55%)	34 (40%)	
Tipo 1	16 (42%)	42 (50%)	
Tipo 2	1 (2,6%)	8 (9,5%)	

* valores expressos como média ± desvio padrão, mediana (25º percentil, 75º percentil) ou n (%). ^†^ p-valor calculado pelo teste t de Student. ^‡^ p-valor calculado pelo teste U de Mann-Whitney. ^§^ p-valor calculado pelo teste de Fisher. SIV: septo interventricular; AE: diâmetro atrial esquerdo; IVAE: índice do volume atrial esquerdo; VE: ventrículo esquerdo; LVEDD: diâmetro diastólico final do ventrículo esquerdo; FEVE: fração de ejeção ventricular esquerda; LVESD: diâmetro sistólico final do ventrículo esquerdo; IVE: índice de massa do ventrículo esquerdo; PP: parede posterior; ERP: espessura relativa de parede.

Apenas os níveis de Pro-BNP e de ureia sérica estavam significativamente mais elevados no grupo não dipper, enquanto o colesterol total estava mais alto no grupo dipper (Tabela 6).

**Tabela 6:** – Resultados laboratoriais divididos nos grupos dippers e não dippers

Variáveis*	Dipper, N = 41	Não dipper, N = 89	p-valor
RLN	1,73 (1,49, 2,46)	1,88 (1,36, 2,44)	0,575^†^
RPL / 1000	61,2 (49,7, 73,8)	60,3 (44,4, 80,7)	0,645^†^
IIS / 1000	473 (378, 572)	444 (314, 639)	0,861^†^
HbA1c (%)	6,0 (5,8, 6,5)	6,3 (5,8, 7,1)	0,196^†^
Ureia (mg/dL)	37 (29, 45)	41 (32, 53)	0,032^†^
Creatinina (mg/dL)	0,97 (0,82, 1,13)	0,96 (0,80, 1,20)	0,867^†^
CLCR (mL/min/1,73 m^2^)	74 (60, 88)	72 (55, 87)	0,462^†^
Colesterol total (mg/dL)	162 (137, 195)	145 (126, 173)	0,036^†^
Ácido úrico (mg/dL)	5,9 (4,6, 6,6)	5,1 (4,4, 6,1)	0,206^†^
Na (mEq/L)	141 (140, 143)	140 (139, 142)	0,225^†^
K (mEq/L)	4,37 (0,50)	4,35 (0,46)	0,851^‡^
ACR (mg/g)	13,4 (8,2, 44,9)	11,8 (5,9, 34,5)	0,600^†^
TSH (µUI/mL)	1,87 (1,25, 2,80)	2,08 (1,52, 3,08)	0,208^†^
Pro-BNP (pg/mL)	55 (28, 87)	80 (45, 163)	0,037^†^

* valores expressos como média ± desvio padrão, mediana (25º percentil, 75º percentil). ^†^ p-valor calculado pelo teste U de Mann-Whitney. ^‡^ p-valor calculado pelo teste t de Student. ACR: razão albumina/creatinina; CLCR: depuração de creatinina; Hb: hemoglobina; HT: hematócrito; RLN: razão neutrófilos/linfócitos; RPL: razão plaquetas/linfócitos; IIS: índice de imunoinflamação sistêmica.

A Tabela 7 apresenta as medições eletrocardiográficas, comparando os grupos dos dippers e não dippers, ajustando para o efeito da idade. No geral, as variáveis eram semelhantes entre os grupos.

**Tabela 7:** – Medições eletrocardiográficas nos grupos dos dippers e não dippers, e a diferença estimada ajustada pela idade entre os grupos

Variáveis*	Descritivas		Inferenciais		
	Dipper, N = 41	Não dipper, N = 89	Diferença ajustada	IC 95%	p-valor^**†**^
FC (bpm)	63,54 (12,88)	64,01 (11,44)	1,1	-3,4, 5,6	0,637
QT-V5 (ms)	422,40 (41,25)	428,76 (49,09)	2,2	-15, 20	0,805
QTc-V5 (ms)	428,29 (40,36)	440,30 (44,99)	14	-3,0, 30	0,108
QTd (ms)	50,29 (32,24)	52,88 (26,49)	0,39	-10, 11	0,941
Tp-Tf V1 (ms)	80,55 (18,20)	76,51 (27,18)	-5,0	-14, 4,4	0,294
Tp-Tf V2 (ms)	79,67 (14,06)	82,90 (25,28)	2,2	-6,3, 11	0,603
Tp-Tf V3 (ms)	78,05 (15,23)	83,13 (25,40)	4,2	-4,4, 13	0,337
Tp-Tf V4 (ms)	79,62 (19,01)	81,32 (28,82)	1,2	-8,9, 11	0,819
Tp-Tf V5 (ms)	76,70 (18,05)	80,47 (23,92)	3,0	-5,5, 12	0,484
Tp-Tf V6 (ms)	76,26 (16,43)	79,08 (24,57)	1,8	-6,9, 10	0,683
Tp-Tf/QT - V1	0,19 (0,04)	0,18 (0,05)	-0,01	-0,03, 0,01	0,443
Tp-Tf/QT - V2	0,19 (0,03)	0,20 (0,05)	0,01	-0,01, 0,02	0,442
Tp-Tf/QT - V3	0,19 (0,04)	0,20 (0,05)	0,01	-0,01, 0,03	0,363
Tp-Tf/QT - V4	0,19 (0,05)	0,19 (0,05)	0,00	-0,02, 0,02	0,900
Tp-Tf/QT - V5	0,18 (0,04)	0,19 (0,04)	0,01	-0,01, 0,02	0,445
Tp-Tf/QT - V6	0,18 (0,03)	0,18 (0,04)	0,00	-0,01, 0,02	0,630
Sokolov (mm)	18,50 (6,36)	16,61 (6,61)	-1,7	-4,2, 0,83	0,189
Cornell (mm)	12,05 (6,94)	12,92 (5,85)	0,41	-1,9, 2,8	0,733
Deflexão intrinsecoide (mm)	47,56 (7,77)	47,32 (9,91)	-0,61	-4,1, 2,9	0,732

* valores expressos como média ± desvio padrão. ^†^ P-valor ajustado pela idade calculado por regressão linear. IC: intervalo de confiança; FC: frequência cardíaca.

Os grupos foram então divididos de acordo com o perfil de hipertensão controlada e não controlada (Tabela 8), e como era esperado, os valores de PA sistólica (PAS) e diastólica (PAD) de 24 horas foram mais elevados no grupo de hipertensão não controlada em comparação com o grupo de hipertensão controlada (135,4 versus 115,1 mmHg, p<0,001 e 82,1 versus 69,1 mmHg, p<0,001), respectivamente.

**Tabela 8:** – Medições eletrocardiográficas nos grupos de hipertensão controlada e não controlada, e a diferença estimada ajustada pela idade entre os grupos

Variáveis*	HA não controlada N=51	HA controlada N=79	Diferença ajustada	IC 95%	p-valor^**†**^
Nº de Anti-hipertensivos	4,29 (1,83)	3,15 (1,26)	-1,1	-1,7 (-0,57)	<0,001
FC (bpm)	64,71 (11,10)	63,32 (12,38)	-1,1	-5,3, 3,2	0,617
QT-V5 (ms)	429,64 (50,91)	424,99 (44,13)	-7,2	-24, 9,2	0,387
QTc-V5 (ms)	440,44 (41,00)	434,14 (45,58)	-5,7	-21, 10	0,480
QTd	52,71 (31,24)	51,65 (26,48)	-2,4	-12, 7,6	0,640
Tp-Tf V1 (ms)	79,94 (30,65)	76,42 (19,64)	-3,9	-13, 5,1	0,392
Tp-Tf V2 (ms)	84,18 (29,43)	80,42 (16,40)	-4,3	-12, 3,7	0,288
Tp-Tf V3 (ms)	85,82 (29,74)	78,74 (16,38)	-7,7	-16, 0,44	0,064
Tp-Tf V4 (ms)	82,37 (34,79)	79,79 (18,83)	-2,9	-12, 6,6	0,541
Tp-Tf V5 (ms)	79,04 (25,83)	79,47 (19,86)	-0,06	-8,1, 8,0	0,987
Tp-Tf V6 (ms)	78,61 (28,70)	77,95 (17,05)	-1,3	-9,3, 6,8	0,759
Tp-Tf/QT - V1	0,19 (0,06)	0,19 (0,04)	0,00	-0,02, 0,01	0,716
Tp-Tf/QT - V2	0,20 (0,05)	0,20 (0,04)	-0,01	-0,02, 0,01	0,440
Tp-Tf/QT - V3	0,20 (0,05)	0,19 (0,04)	-0,01	-0,03, 0,00	0,066
Tp-Tf/QT - V4	0,19 (0,06)	0,19 (0,04)	-0,01	-0,02, 0,01	0,521
Tp-Tf/QT - V5	0,18 (0,04)	0,19 (0,04)	0,00	-0,01, 0,02	0,555
Tp-Tf/QT - V6	0,18 (0,05)	0,18 (0,04)	0,00	-0,01, 0,02	0,774
Sokolov (mm)	19,92 (7,46)	15,41 (5,24)	-4,4	-6,6, -2,2	<0,001
Cornell (mm)	12,55 (7,16)	12,72 (5,53)	-0,07	-2,3, 2,1	0,953
Deflexão intrinsecoide (mm)	49,18 (8,22)	46,23 (9,75)	-3,2	-6,5, 0,10	0,057

* valores expressos como média ± desvio padrão. ^†^ P-valor ajustado pela idade calculado por regressão linear. FC: frequência cardíaca; HA: hipertensão arterial; IC: intervalo de confiança.

Os pacientes também foram divididos nos grupos com hipertensão resistente e não resistente (Tabela 9).

**Tabela 9:** – Medições eletrocardiográficas nos grupos de hipertensão resistente e não resistente, e a diferença estimada ajustada pela idade entre os grupos

Variáveis*	Resistente N=62	Não resistente N=68	Diferença ajustada	IC 95%	p-valor^**†**^
PAS total (mmHg)	128,7 (14,0)	118,3 (10,3)	11,1	6,8, 15,3	<0,001
PAD total (mmHg)	77,0 (10,3)	71,7 (8,8)	4,7	1,4, 8,0	0,006
Nº de Anti-hipertensivos	4,76 (1,28)	2,54 (1,04)	2,2	1,8, 2,6	<0,001
FC (bpm)	63,97 (12,74)	63,76 (11,11)	-0,28	-4,5, 3,9	0,896
QT-V5 (ms)	433,30 (50,53)	420,96 (42,58)	16	0,33, 32	0,046
QTc-V5 (ms)	443,91 (49,32)	430,00 (37,35)	13	-2,1, 29	0,091
QTd	52,94 (28,20)	51,26 (28,63)	3,6	-6,2, 13	0,469
Tp-Tf V1 (ms)	81,09 (28,55)	74,89 (20,15)	7,3	-1,5, 16	0,105
Tp-Tf V2 (ms)	85,42 (26,95)	78,78 (16,99)	7,8	0,02, 16	0,049
Tp-Tf V3 (ms)	84,65 (28,04)	78,73 (16,40)	6,9	-1,1, 15	0,090
Tp-Tf V4 (ms)	84,98 (33,37)	77,15 (16,88)	8,5	-0,68, 18	0,069
Tp-Tf V5 (ms)	84,46 (26,16)	74,68 (16,98)	11	3,1, 18	0,006
Tp-Tf V6 (ms)	81,48 (27,41)	75,20 (16,01)	7,4	-0,49, 15	0,066
Tp-Tf/QT - V1	0,19 (0,05)	0,18 (0,04)	0,01	-0,01, 0,03	0,188
Tp-Tf/QT - V2	0,20 (0,05)	0,19 (0,04)	0,02	0,00 (0,03)	0,050
Tp-Tf/QT - V3	0,20 (0,05)	0,19 (0,04)	0,01	0,00 (0,03)	0,069
Tp-Tf/QT - V4	0,20 (0,06)	0,18 (0,04)	0,01	0,00 (0,03)	0,090
Tp-Tf/QT - V5	0,19 (0,05)	0,18 (0,04)	0,02	0,00 (0,03)	0,019
Tp-Tf/QT - V6	0,19 (0,05)	0,18 (0,03)	0,01	-0,01, 0,02	0,200
Sokolov (mm)	18,84 (6,81)	15,72 (6,01)	3,0	0,70 (5,2)	0,011
Cornell (mm)	14,20 (7,18)	11,26 (4,80)	3,4	1,3 (5,5)	0,002
Deflexão intrinsecoide (mm)	47,63 (9,54)	47,18 (9,06)	0,76	-2,5, 4,0	0,647

* valores expressos como média ± desvio padrão. ^†^ P-valor ajustado pela idade calculado por regressão linear. FC: frequência cardíaca; IC: intervalo de confiança; PAS: pressão arterial sistólica; PAD: pressão arterial diastólica.

Para avaliar a reprodutibilidade intraobservador das medições, os intervalos QT e Tp-Tf foram selecionados para serem retestados em 10% da amostra total; ou seja, 13 ECGs foram selecionados aleatoriamente e reanalisados pelo pesquisador após todas as medições do estudo serem concluídas.

Para o intervalo QT, o erro absoluto médio foi de 0,88 ms (DP ± 7,33 ms), representando um erro relativo de 1,46%, IC 95% de -15,2 a +13,5 ms. Para a medição do intervalo Tp-Tf, o erro absoluto foi de 0,29 ms (DP ± 5,99 ms), representando um erro relativo de 5,91%, IC 95% de -12,0 a +11,4 ms.

## Discussão

Em nosso estudo, o perfil dos pacientes consistiu em indivíduos hipertensos com alto risco cardiovascular. Em comparação com o estudo de Demir e Uyan,^12^ que serviu como base para o cálculo da amostra deste estudo, foram excluídos pacientes com diabetes mellitus, doença renal crônica e apneia obstrutiva do sono. Nesse estudo, apenas 63% dos pacientes estavam utilizando terapia combinada, em comparação com 94% em nosso estudo.

Esses mesmos critérios de exclusão foram aplicados por Karaagac et al.^13^ Além disso, o perfil de alto risco cardiovascular e a idade mais avançada em nosso estudo, em comparação com outros estudos, podem explicar a alta prevalência de não dippers (68%) na população estudada.

Não encontramos diferenças significativas entre as medições eletrocardiográficas da repolarização ventricular e da hipertrofia ventricular esquerda (HVE) ao comparar os grupos dippers e não dippers.

Demir e Uyan^12^ estudaram 80 pacientes hipertensos (30 não dippers), com uma idade média de 51,5 ± 8 anos para os dippers e 50,6 ± 5,4 anos para os não dippers. Os intervalos Tp-Tf, a razão Tp-Tf/QT e o QTd foram significativamente mais elevados nos não dippers. Eles também demonstraram uma associação com o aumento dos índices de massa ventricular esquerda medidos por ecocardiografia.

No entanto, ao contrário de nosso estudo, Demir e Uyan^12^ excluíram pacientes com ondas U e mediram o ponto final da onda T quando a porção descendente tocava a linha isoelétrica, sem traçar uma tangente. Eles também não especificaram quais derivações representavam seus resultados.

Karaagac et al.^13^ avaliaram 70 pacientes hipertensos com síndrome metabólica, com uma idade média de 55 ± 11 anos (35 não dippers). Na derivação DII, eles encontraram um aumento significativo nos intervalos Tp-Tf (91 vs. 74 ms; p <0,001), Tp-Tf/QT (0,24 vs. 0,20 ms, p <0,001) e Tp-Tf/QTc (0,22 vs. 0,18 ms, p <0,001) nos não dippers. No entanto, essa associação não foi observada para os intervalos QT e QTc ou para as medições ecocardiográficas.

Um estudo maior de Yan et al.^14^ selecionou 171 pacientes - 102 idosos (>60 anos) e 69 mais jovens ou de meia-idade (<60 anos) com HA tratada. Apenas nos pacientes <60 anos houve um aumento no QTc nos não dippers (438 ms vs. 416 ms, p <0,05), sem diferença no Tp-Tf. No grupo >60 anos, não foram encontradas diferenças significativas entre os grupos de dipper, não dipper e dipper reverso.

Em um estudo notável, Passino et al.^15^ selecionaram 48 pacientes hipertensos primários não tratados, dos quais 20 foram classificados como não dippers, com uma idade média de aproximadamente 46 anos e uma duração média de diagnóstico de 1,2 anos. Este estudo avaliou parâmetros eletrocardiográficos ao longo de 24 horas, encontrando intervalos QT e QTc significativamente mais elevados nos não dippers durante o sono, enquanto nenhuma diferença significativa foi observada no período diurno.

Esses achados demonstram a falta de consenso na literatura e a ausência de padronização na medição do ponto final da onda T entre os estudos. Também é razoável considerar que as alterações na repolarização ventricular devido à redução anormal da pressão noturna possam ser mais evidentes durante o sono do que durante a vigília.

Além disso, as diferenças nas medidas de repolarização ventricular entre os grupos dipper e não dipper em nosso estudo podem ter sido atenuadas pela idade avançada dos pacientes e pelo maior perfil de comorbidades, conforme demonstrado por Yan et al.^14^

Em nosso estudo, embora tenhamos encontrado um aumento estatisticamente significativo nos níveis de pro-BNP no perfil não dipper, esses valores não puderam ser associados à insuficiência cardíaca com fração de ejeção reduzida, pois a FEVE <50% foi um critério de exclusão para o estudo. Os níveis de pro-BNP poderiam estar associados a uma maior prevalência de insuficiência cardíaca com fração de ejeção preservada. Entretanto, no ecocardiograma em repouso, esse resultado também não refletiu diferenças ecocardiográficas significativas ao comparar os grupos dipper e não dipper.

Isso se alinha a outros achados na literatura, apesar do debate contínuo. Por exemplo, Cuspidi et al.^16^ estudaram 111 pacientes hipertensos com diagnóstico recente, dos quais 33 eram não dippers, e não encontraram diferenças significativas em HVE e espessura de carótida entre os grupos.

Roman et al.^17^ avaliaram 183 pacientes hipertensos (79 não dippers) e observaram uma prevalência maior de placas na carótida nos não dippers (41% vs. 27%). No entanto, essa significância foi perdida quando ajustada para a idade, sem diferenças significativas na estrutura ventricular esquerda e na função sistólica entre os grupos.

Ferrara et al.^18^ estudaram 179 pacientes hipertensos (123 hipertensos de longa data e 56 recém-diagnosticados) e não encontraram diferença nas medições ecocardiográficas de disfunção diastólica, massa ventricular esquerda ou diâmetro atrial esquerdo entre os grupos dipper e não dipper.

Uma metanálise de 19 estudos também não encontrou uma correlação significativa entre a redução da pressão arterial noturna e a HVE.^19^

Em contraste, em um estudo chinês de centro único, um grupo de 183 pacientes hipertensos, independentemente do tratamento, com uma idade média de 46,5 anos e duração da hipertensão de 5 anos, incluiu 117 não dippers. Esse estudo demonstrou um aumento significativo na remodelação ventricular em pacientes não dippers. Esses resultados foram demonstrados por um aumento no índice de massa ventricular esquerda (iVE) (101,3 g/m^2^ no grupo não-dipper, comparado a 92,3 g/m^2^ no grupo dipper, p=0,029), associado a taxas mais altas de índice de massa ventricular esquerda, resultando em geometria ventricular esquerda anormal em 73,5% do grupo não dipper, comparado a 51,5% do grupo dipper (p=0,001).^20^

Resultados ainda mais divergentes foram obtidos por Rodrigues et al.^21^ em um estudo de ressonância magnética que avaliou 99 pacientes hipertensos, incluindo 35 não dippers. Nesse estudo, o grupo não dipper não foi associado a um aumento na massa ventricular quando comparado ao grupo dipper. No entanto, aqueles classificados como dippers extremos (queda da PAS noturna >20%) apresentaram um aumento significativo na massa ventricular indexada e hipertrofia ventricular concêntrica.

Embora os primeiros estudos publicados entre 1988 e 1997 tenham demonstrado uma associação entre a queda da PA noturna e danos aos órgãos, como doença da artéria carótida, HVE e até acidente vascular cerebral,^22-26^ estudos maiores e mais contemporâneos não conseguiram reproduzir esses resultados. Isso levantou questionamentos sobre se o padrão não dipper, como marcador de prognóstico desfavorável para eventos cardiovasculares, está relacionado à hipertensão noturna ou a outros efeitos, como disfunção autonômica, maior prevalência de DRC, diabetes mellitus, síndrome da apneia-hipopneia obstrutiva do sono e hipertensão secundária.

A maior prevalência do uso de metildopa no grupo de pacientes dipper pode ser explicada pelo seu efeito farmacológico como bloqueador alfa-adrenérgico, considerando que a estimulação adrenérgica é um mecanismo muito importante para a queda anormal da pressão arterial durante a noite, especialmente em pacientes com SAHOS. O uso de bloqueadores alfa-adrenérgicos tem sido associado à melhora da pressão arterial noturna em comparação com diuréticos tiazídicos.^27^

A análise secundária em nosso estudo mostrou que, ao classificar os pacientes com base na resistência da hipertensão, o grupo resistente apresentou aumentos significativos nos intervalos QT, Tp-Tf e Tp-Tf/QT, além de valores mais altos nos índices de Sokolow-Lyon e Cornell (Figura Central). Esses resultados não parecem estar associados a diferenças na PA sistólica (PAS) e diastólica (PAD), já que achados semelhantes não foram observados entre os grupos com HA controlada e não controlada.

Este é o primeiro estudo publicado que demonstra uma associação entre HA resistente e alterações na repolarização ventricular, um achado clinicamente relevante. Salles et al. (2009), em um estudo de coorte prospectivo com 538 pacientes com HA resistente, encontraram que o QTc prolongado estava associado a um aumento do risco de mortalidade cardiovascular e global durante um seguimento mediano de 4,8 anos.^28^

As limitações do nosso estudo incluem:

1. A natureza unicêntrica;
2. A análise eletrocardiográfica ser realizada apenas durante o período de vigília;
3. A proporção de pacientes dipper e não dipper, o que pode contribuir para a redução do poder dos resultados;
4. A falta de um MAPA de 24 horas ou em dias alternados para uma melhor classificação dos grupos;^29,30^
5. Em nosso estudo, o critério fundamental para inclusão foi a realização recente de um exame de monitorização ambulatorial da pressão arterial (MAPA), o que pode introduzir viés de seleção, considerando que os critérios utilizados para indicar o MAPA na prática clínica incluem pacientes com suspeita de efeito do jaleco branca, hipertensão mascarada, hipertensão resistente, pacientes com sintomas sugestivos de hipotensão, entre outros;
6. A medição da repolarização ventricular realizada exclusivamente pelo método da tangente, ao contrário de outros estudos que utilizaram o método da cauda, embora a literatura atual sugira o método da tangente como o mais apropriado.

Por fim, esperamos que os dados encontrados neste estudo possam auxiliar e contribuir para a literatura, para que possamos entender a real associação entre a queda da pressão arterial noturna e as medições de repolarização ventricular.

## Conclusão

Este foi o primeiro estudo realizado com o objetivo de analisar a repolarização ventricular em pacientes hipertensos dippers e não dippers com este perfil etário e comorbidades, indicando um perfil de alto risco cardiovascular, com alta prevalência de não dippers.

Nessa população, não encontramos diferenças significativas nas características clínicas, demográficas e nos parâmetros ecocardiográficos ao comparar pacientes dippers e não dippers.

Em relação aos parâmetros eletrocardiográficos de repolarização ventricular e hipertrofia ventricular esquerda, ao medir o intervalo QT, intervalo QTc, Tp-Tf e Tp-Tf/QT pelo método da tangente, não encontramos diferenças entre dippers e não dippers.

Entretanto, em pacientes com hipertensão resistente, foi observado um aumento significativo nas medições de repolarização ventricular. Para esse grupo de pacientes, a implementação de eletrocardiogramas na rotina de pacientes ambulatoriais poderia ser uma ferramenta útil para a estratificação do risco de desfechos cardiovasculares.
