# Supplementary material for: Análise da Repolarização Ventricular em Pacientes Hipertensos: Influência do Fenômeno de Redução da Pressão Arterial durante a Noite
Source: Arq Bras Cardiol. 2025 Apr 29;122(4):e20240725. [Article in Portuguese] doi: 10.36660/abc.20240725 (PMC12108120; doi:10.36660/abc.20240725)
Supplement: Translation

# Translation: Analysis of Ventricular Repolarization in Hypertensive Patients: Influence of Nocturnal Blood Pressure Dipping

**DOI:** 10.36660/abc.20240725i

**Authors:** Marco Aurélio Goulart, Dalmo Antonio Ribeiro Moreira, Fernando Yue Cesena, Jonathan Batista Souza, Antonio Gabriele Laurinavicius, Fernanda Marciano Consolim-Colombo, Márcio Gonçalves de Sousa

**Affiliations:** 1 Brazil Instituto Dante Pazzanese de Cardiologia, São Paulo, SP – Brazil; 2 Brazil Instituto do Coração do Hospital das Clínicas da Faculdade de Medicina da Universidade de São Paulo, São Paulo, SP – Brazil

**Keywords:** Hypertension, Heart Disease Risk Factors, Cardiac Electrophysiology

## Abstract

Changes in ventricular repolarization are associated with ventricular arrhythmias and higher mortality. The association between a non-dipper blood pressure pattern and changes in ventricular repolarization remains controversial. This study sought to compare ventricular repolarization measurements (QT interval, QTc, Tp-Te, Tp-Te/QT, and QTd) in hypertensive dippers and non-dippers. Secondary objectives are to compare measurements between controlled and uncontrolled hypertensive patients, as well as resistant and non-resistant hypertensive patients. This observational, cross-sectional study involved patients monitored in a Hypertension Service. The level of significance adopted in the statistical analysis was 5%. A total of 130 participants were admitted. The mean age was 67.4 years, with 72% presenting some form of target organ damage. Repolarization measurements did not differ between dippers and non-dippers. However, within the resistant hypertension group, when compared to the non-resistant, differences were observed in the QT interval in V5 (433.3 ms vs. 420.9 ms, p = 0.046), Tp-Te in both V2 (85.4 ms vs. 78.7 ms, p = 0.049) and V5 (84.6 ms vs. 74.6 ms, p = 0.006), Tp-Te/QT in V5 (0.19 vs. 0.18, p = 0.019), Sokolow-Lyon index (18.8 mm vs. 15.7 mm, p = 0.011), and Cornell index (14.2 mm vs. 11.2 mm, p = 0.002), aged-adjusted values. In this high cardiovascular risk hypertensive population, no difference in repolarization measures was found between dippers and non-dippers. However, this is the first study to demonstrate increased ventricular repolarization measures in patients with resistant hypertension.

## Introduction

Changes in the ventricular repolarization period play an important role in the formation of ventricular arrhythmias and are associated with higher mortality. QT and QTc intervals, especially measured in DII and V5, represent a result of ventricular repolarization.^1-3^

QT dispersion (QTd), which is the difference between the longest and shortest QT interval among the 12 leads, aims to estimate the time difference of ventricular repolarization across various regions of the heart.^4,5^ The Tp-Te interval (Tpeak to Tend) represents transmural repolarization involving the epicardium, endocardium, and mesocardium.^6-9^

In patients with arterial hypertension (AH), changes in ventricular repolarization arise from electrical remodeling caused by cardiomyocyte hypertrophy, collagen deposition, and myocardial ischemia due to an imbalance between supply and demand.^10,11^ However, in blood pressure non-dipper patients, the association with ventricular repolarization changes continues to be controversial.^12-14^

This study aims to compare five distinct measures of ventricular repolarization (QT interval, QTc, Tp-Te, Tp-Te/QT, and QTd) by comparing groups of hypertensive dipper and non-dipper patients. Secondary objectives include comparing ventricular repolarization measures when dividing patients into controlled and uncontrolled hypertensive patients, as well as resistant and non-resistant hypertensive patients.

## Methods

### Study design and population

This is an observational, cross-sectional study involving hypertensive patients monitored in the Hypertension Department of a tertiary hospital, who underwent ambulatory blood pressure monitoring (ABPM). Through this examination, dipper and non-dipper patients were selected.

Based on a standard deviation of 11.2 ms for the Tp-Te variable identified in the study by Demir and Uyan,^12^ an N of 130 patients was estimated to detect differences of up to 8 ms between groups, with a 90% power and a 5% significance level.

Exclusion criteria included patients who had ABPM with <70% of valid measurements, stage 3 AH defined by systolic blood pressure (SBP) ≥ 180 or diastolic blood pressure (DBP) ≥ 110 mmHg), cardiomyopathies (hypertrophic, ischemic, congenital, or valvular with moderate or important dysfunction), left ventricular dysfunction below 50%, diagnosed obstructive coronary artery disease, pacemaker carriers, patients on hemodialysis, severe liver disease, pregnancy, secondary AH, active neoplasia under treatment, BMI > 34.9, atrial fibrillation or flutter, use of amiodarone, propafenone, sotalol, verapamil or diltiazem, non-interpretable electrocardiogram for other reasons, and individuals already participating in other research.

### Data collection

The ABPM exam was conducted using the DYNA-MAPA NG device, with measurements taken every 20 minutes during the day and every 30 minutes at night. The sleep period was based on the event report filled out by the patient.

Patients with a reduction of less than 10% in SBP and/or DBP during sleep compared to wake were classified as non-dippers. Uncontrolled AH was defined as SBP ≥ 130 and/or DBP ≥ 80 mmHg based on ABPM.

Patients were considered to have resistant hypertension if: 1)vABPM revealed systolic SBP ≥ 130 mmHg and/or DBP ≥ 80 mmHg despite optimized therapy with a calcium channel blocker (CCB), thiazide diuretic, and either an angiotensin receptor blocker (ARB) or angiotensin-converting enzyme inhibitor (ACEI); or 2) ABPM revealed SBP <130 mmHg and DBP < 80 mmHg while using optimized doses of CCB, thiazide diuretic, and either an ARB or ACEI, combined with a fourth class of antihypertensive agents.

The 12-lead electrocardiogram was performed using the Eletro System PRE0900001 device, standardized with a paper speed of 25 mm/s and amplitude of 10 mm/mV. The measurements were conducted by the researcher using semi-automatic assistance from the Eletro System V6.4.0.11 software.

The QT and QTc intervals were measured in all 12 leads, but only V5 was used for representation. The Tp-Te interval was measured across six leads in the horizontal plane. Measurements were performed using the tangent method, where a tangent line was drawn on the steepest slope of the last segment of the T wave until it intersected with the baseline.

QT interval correction for heart rate was performed using Bazzett’s formula, applying the RR interval between the measured complex and the previous one. The Sokolow-Lyon index was measured by adding the S wave amplitude in V1 to the highest R wave amplitude found in V5 or V6. Cornell’s criterion was established by adding the R wave amplitude in lead aVL to the S wave amplitude in lead V3.

The Epworth Sleepiness Scale (ESS) questionnaire and neck circumference measurements were collected by the researcher.

### Statistical analysis

Continuous variables were presented as means ± standard deviations or medians and interquartile ranges, as appropriate. Categorical variables were presented as absolute and relative frequencies. Group comparisons were performed using the unpaired Student’s t-test (continuous variables with normal distribution), the Mann-Whitney *U* test (continuous variables with non-normal distribution), or the Fisher’s test (categorical variables). Normality of data distribution was assessed by the Anderson-Darling test along with visual inspection of histograms and quantile-quantile-plots.

The association of a non-dipper or dipper status with electrocardiographic variables was evaluated using linear regression models adjusted for age. In these models, the dependent variable was the electrocardiographic feature (e.g., heart rate), the independent variable was the non-dipper/dipper phenotype, and age was a covariate. Estimates, along with 95% confidence intervals and p-values, were reported corresponding to the age-adjusted differences in the dependent variables between the non-dipper and dipper groups. The same procedure was performed to estimate age-adjusted differences in electrocardiographic measurements between participants with controlled versus uncontrolled hypertension, as well as between individuals with or without resistant hypertension.

The level of significance adopted in the statistical analysis was 5%. Statistical analyses were performed using the R software (version 4.1.2) and the *jamovi* software (version 2.3.26.0). This study followed the principles of the Declaration of Helsinki and Nuremberg Code, in compliance with Brazil’s National Health Council Research Standards involving Human Subjects (Res. CNS 196/96). It was approved by the Research Ethics Committee under opinion number 6.213.951, and all patients or their legal representatives signed the free and informed consent form.

## Results

A total of 130 participants were admitted to the study, with a median time of 3.8 weeks between the EKG and ABPM exams. Table 1 shows the proportion of patients classified as dippers and non-dippers based on ABPM results.

**Table 1:** – Blood pressure values obtained by ABPM comparing groups of dippers and non-dippers

Variables*	Dipper, N = 41	Non-dipper, N = 89	p-value
**24h SBP (mmHg)**	124.3 (12.2)	122.8 (13.7)	0.534^†^
**24h DBP (mmHg)**	75.6 (10.3)	73.6 (9.7)	0.291^†^
**Wake SBP (mmHg)**	128.6 (12.4)	123.7 (13.4)	0.042^†^
**Wake DBP (mmHg)**	79.2 (10.4)	75.1 (10.1)	0.035^†^
**Sleep SBP (mmHg)**	111.7 (11.5)	120.4 (16.2)	<0.001^†^
**Sleep DBP (mmHg)**	64.2 (9.8)	69.5 (9.9)	0.005^†^
**SBP Dipping (%)**	12 (11, 14)	4 (-1, 7)	<0.001^§^
**DBP Dipping (%)**	19 (15, 22)	7 (-1, 10)	<0.001^§^
**Uncontrolled AH (%)**	19 (49%)	31 (35%)	0.151^‡^

* values expressed as mean ± standard deviation, median (25th percentile, 75th percentile) or n (%). ^†^ p-value calculated by the Student’s t test. ^‡^ p-value calculated by the Fisher’s test. ^§^ p-value calculated by the Mann-Whitney U test. AH: arterial hypertension; DBP: diastolic blood pressure; SBP: systolic blood pressure.

Table 2 shows a tendency for higher age in the non-dipper group, although without statistical significance. Other characteristics also did not present statistically significant differences.

**Table 2:** – Demographic, anthropometric, and clinical characteristics in hypertensive patients, divided into dipper and non-dipper groups

Variables*	Dipper, N = 41	Non-dipper, N = 89	p-value
**Age (years)**	66 (59. 73)	71 (64. 74)	0.095^§^
**Female Gender (%)**	27 (66%)	62 (70%)	0.688^‡^
**Race (n/%)**			0.418^‡^
White	23 (56%)	60 (67%)	
Brown	6 (15%)	12 (13%)	
Black	12 (29%)	17 (19%)	
**BMI**	30.0 (5.0)	29.3 (4.9)	0.476^†^
**Duration of AH (years)**	26.0 (16.5, 38.5)	28.0 (22.0, 39.2)	0.348^§^
**Epworth ES (pts)**	7 (4, 15)	5 (2, 12)	0.289^§^
**Neck Circumference (cm)**	36 (35, 39)	37 (35, 40)	0.419^§^
Man	40.7	41.4	
Woman	34.8	35.8	

* values expressed as mean± standard deviation, median (25th percentile, 75th percentile), or n (%). ^†^ p-value calculated by the Student’s t test. ^‡^ p-value calculated by the Fisher’s test. ^§^ p-value calculated by the Mann-Whitney U test. AH: arterial hypertension; BMI: body mass index; ES: sleepiness scale.

Table 3 demonstrates a high cardiovascular risk profile, with dyslipidemia and prediabetes/diabetes present in the majority of patients, as well as at least one target organ lesion caused by AH, with no significant difference between groups.

**Table 3:** – Distribution of comorbidities in hypertensive patients, divided into dipper and non-dipper groups

Variables*	Dipper, N = 41	Non-dipper, N = 89	p-value
**Diabetes/Prediabetes**	34 (83%)	80 (90%)	0.265
**Stroke**	5 (12%)	14 (16%)	0.790
**Dyslipidemia**	38 (93%)	86 (97%)	0.379
**Current/Former Smoker**	18 (44%)	28 (31%)	0.174
**Chronic Kidney Disease**	11 (27%)	28 (31%)	0.683
**Target Organ Damage**	26 (63%)	67 (75%)	0.210
LVH	19 (46%)	41 (46%)	>0.999
Microalbuminuria	12 (29%)	30 (34%)	0.689
Hypertensive Retinopathy	13/32 (41%)	38/81 (47%)	0.675

* values expressed as n (%). ^†^ p-value calculated by the Fisher’s test. LVH: left ventricular hypertrophy.

The average number of antihypertensive drug classes used was three, with no difference between groups, except for a higher prevalence of central sympatholytic use (represented by methyldopa) in the dipper group (Table 4).

**Table 4:** – Distribuição de medicamentos dividida nos grupos dippers e não dippers

Variables*	Dipper, N = 41	Non-dipper, N = 89	p-value
Nº of Antihypertensives	4 (3, 5)	3 (3, 4)	0.427^†^
ACEI/ARB	39 (95%)	84 (94%)	>0.999^‡^
CCB	34 (83%)	68 (76%)	0.494^‡^
Thiazide Diuretic	33 (80%)	65 (73%)	0.391^‡^
Loop Diuretic	1 (2.4%)	7 (7.9%)	0.434^‡^
Aldosterone Antagonist	16 (39%)	25 (28%)	0.229^‡^
Clonidine	2 (4.9%)	7 (7.9%)	0.719^‡^
Hydralazine	3 (7.3%)	5 (5.6%)	0.707^‡^
Nitrate	2 (4.9%)	2 (2.2%)	0.590^‡^
Doxazosin	1 (2.4%)	2 (2.2%)	>0.999^‡^
Beta-Blocker	20 (49%)	46 (52%)	0.851^‡^
Methyldopa	6 (15%)	1 (1.1%)	0.004^‡^

* values expressed as median (25th percentile, 75th percentile) or n (%). ^†^ p-value calculated by the Mann-Whitney U test. ^‡^ p-value calculated by the Fisher’s test. ACEI: angiotensin-converting enzyme inhibitor; ARB: angiotensin receptor blocker; CCB: calcium channel blocker.

There were no differences in echocardiographic measures between the groups. However, it is important to note that the median value of left atrial volume index (LAVi ml/m^2^) was above the reference value of 34 ml/m^2^ in both groups (Table 5).

**Table 5:** – Echocardiographic measurements in hypertensive patients, divided into dipper and non-dipper groups

Variables*	Dipper, N = 41	Non-dipper, N = 89	p-value
**LA Diameter (mm)**	39.2 (4.5)	39.2 (5.0)	0.951^†^
**LAVi (ml/m^2^)**	35 (30, 38)	35 (32, 40)	0.370^‡^
**LVEDD (mm)**	48 (45, 53)	47 (43, 51)	0.235^‡^
**LVESD (mm)**	31 (28, 33)	31 (28, 33)	0.691^‡^
**IVS Diameter (mm)**	10 (9, 11)	10 (9, 10)	0.134^‡^
**PW Diameter (mm)**	9 (9, 10)	9 (9, 10)	0.548^‡^
**LV Mass (g)**	170 (134, 194)	150 (128, 186)	0.171^‡^
**LV Mass Index (g/m^2^)**	90.7 (22.9)	89.5 (20.2)	0.775^†^
**RWT**	0.38 (0.35, 0.42)	0.38 (0.35, 0.42)	0.765^‡^
**Diastolic dysfunction**			0.206^§^
None	21 (55%)	34 (40%)	
Type 1	16 (42%)	42 (50%)	
Type 2	1 (2,6%)	8 (9,5%)	

* values expressed as mean ± standard deviation, median (25th percentile, 75th percentile), or n (%). ^†^ p-value calculated by the Student’s t test. ^‡^ p-value calculated by the Mann-Whitney U test. ^§^ p-value calculated by the Fisher’s test. IVS: interventricular septum; LA: left atrium diameter; LAVi: left atrial volume index; LV: left ventricle; LVEDD: left ventricular end-diastolic diameter; LVEF: left ventricular ejection fraction; LVESD: left ventricular end-systolic diameter; LVi: left ventricle mass index; PW: posterior wall; RWT: relative wall thickness.

Only Pro-BNP and serum urea levels were significantly higher in the non-dipper group, while total cholesterol was higher in the dipper group (Table 6).

**Table 6:** – Laboratory results divided by dipper and non-dipper groups

Variables*	Dipper, N = 41	Non-dipper, N = 89	p-value
NLR	1.73 (1.49, 2.46)	1.88 (1.36, 2.44)	0.575^†^
PLR / 1000	61.2 (49.7, 73.8)	60.3 (44.4, 80.7)	0.645^†^
SII / 1000	473 (378, 572)	444 (314, 639)	0.861^†^
HbA1c (%)	6.0 (5.8, 6.5)	6.3 (5.8, 7.1)	0.196^†^
Urea (mg/dL)	37 (29, 45)	41 (32, 53)	0.032^†^
Creatinine (mg/dL)	0.97 (0.82, 1.13)	0.96 (0.80, 1.20)	0.867^†^
CrCl (mL/min/1,73 m^2^)	74 (60, 88)	72 (55, 87)	0.462^†^
Total Cholesterol (mg/dL)	162 (137, 195)	145 (126, 173)	0.036^†^
Uric Acid (mg/dL)	5.9 (4.6, 6.6)	5.1 (4.4, 6.1)	0.206^†^
Na (mEq/L)	141 (140, 143)	140 (139, 142)	0.225^†^
K (mEq/L)	4.37 (0.50)	4.35 (0.46)	0.851^‡^
ACR (mg/g)	13.4 (8.2, 44.9)	11.8 (5.9, 34.5)	0.600^†^
TSH (µUI/mL)	1.87 (1.25, 2.80)	2.08 (1.52, 3.08)	0.208^†^
Pro-BNP (pg/mL)	55 (28, 87)	80 (45, 163)	0.037^†^

* values expressed as mean ± standard deviation or median (25th percentile, 75th percentile). ^†^ p-value calculated by the Mann-Whitney U test. ^‡^ p-value calculated by the Student’s t test. ACR: albumin/creatinine ratio; CrCl: creatinine clearance; Hb: hemoglobin; HT: hematocrit; NLR: neutrophil/lymphocyte ratio; PLR: platelet/lymphocyte ratio; SII: systemic immunoinflammation index.

Table 7 presents electrocardiographic measurements, comparing the dipper and non-dipper groups while adjusting for the effect of age. Overall, the variables were similar between groups.

**Table 7:** – Electrocardiographic measurements in the dipper and non-dipper groups, and the estimated age-adjusted difference between the groups

Variables*	Descriptive		Inferential		
	Dipper, N = 41	Non-dipper, N = 89	Adjusted difference	95% CI	p-value^**†**^
HR (bpm)	63.54 (12.88)	64.01 (11.44)	1.1	-3.4, 5.6	0.637
QT-V5 (ms)	422.40 (41.25)	428.76 (49.09)	2.2	-15, 20	0.805
QTc-V5 (ms)	428.29 (40.36)	440.30 (44.99)	14	-3.0, 30	0.108
QTd (ms)	50.29 (32.24)	52.88 (26.49)	0.39	-10, 11	0.941
Tp-Te V1 (ms)	80.55 (18.20)	76.51 (27.18)	-5.0	-14, 4.4	0.294
Tp-Te V2 (ms)	79.67 (14.06)	82.90 (25.28)	2.2	-6.3, 11	0.603
Tp-Te V3 (ms)	78.05 (15.23)	83.13 (25.40)	4.2	-4.4, 13	0.337
Tp-Te V4 (ms)	79.62 (19.01)	81.32 (28.82)	1.2	-8.9, 11	0.819
Tp-Te V5 (ms)	76.70 (18.05)	80.47 (23.92)	3.0	-5.5, 12	0.484
Tp-Te V6 (ms)	76.26 (16.43)	79.08 (24.57)	1.8	-6.9, 10	0.683
Tp-Te/QT - V1	0.19 (0.04)	0.18 (0.05)	-0.01	-0.03, 0.01	0.443
Tp-Te/QT - V2	0.19 (0.03)	0.20 (0.05)	0.01	-0.01, 0.02	0.442
Tp-Te/QT - V3	0.19 (0.04)	0.20 (0.05)	0.01	-0.01, 0.03	0.363
Tp-Te/QT - V4	0.19 (0.05)	0.19 (0.05)	0.00	-0.02, 0.02	0.900
Tp-Te/QT - V5	0.18 (0.04)	0.19 (0.04)	0.01	-0.01, 0.02	0.445
Tp-Te/QT - V6	0.18 (0.03)	0.18 (0.04)	0.00	-0.01, 0.02	0.630
Sokolov (mm)	18.50 (6.36)	16.61 (6.61)	-1.7	-4.2, 0.83	0.189
Cornell (mm)	12.05 (6.94)	12.92 (5.85)	0.41	-1.9, 2.8	0.733
Intrinsicoid Deflection (mm)	47.56 (7.77)	47.32 (9.91)	-0.61	-4.1, 2.9	0.732

* values expressed as mean ± standard deviation. † Age-adjusted p-value calculated by linear regression. CI: confidence interval; HR: Heart rate.

The groups were then divided according to the profile of controlled and uncontrolled hypertension (Table 8), and as expected, the 24-hour SBP and DBP values were higher in the uncontrolled hypertension group, when compared to the controlled hypertension group (135.4 versus 115.1 mmHg, p<0.001 and 82.1 versus 69.1 mmHg, p<0.001), respectively.

**Table 8:** – Electrocardiographic measurements in the groups with controlled and uncontrolled hypertension, and the estimated age-adjusted difference between the groups

Variables*	Uncontrolled AH N=51	Controlled AH N=79	Adjusted difference	95% CI	p-value^**†**^
No. of Antihypertensives	4.29 (1.83)	3.15 (1.26)	-1.1	-1.7, -0.57	<0.001
HR (bpm)	64.71 (11.10)	63.32 (12.38)	-1.1	-5.3, 3.2	0.617
QT-V5 (ms)	429.64 (50.91)	424.99 (44.13)	-7.2	-24, 9.2	0.387
QTc-V5 (ms)	440.44 (41.00)	434.14 (45.58)	-5.7	-21, 10	0.480
QTd	52.71 (31.24)	51.65 (26.48)	-2.4	-12, 7.6	0.640
Tp-Te V1 (ms)	79.94 (30.65)	76.42 (19.64)	-3.9	-13, 5.1	0.392
Tp-Te V2 (ms)	84.18 (29.43)	80.42 (16.40)	-4.3	-12, 3.7	0.288
Tp-Te V3 (ms)	85.82 (29.74)	78.74 (16.38)	-7.7	-16, 0.44	0.064
Tp-Te V4 (ms)	82.37 (34.79)	79.79 (18.83)	-2.9	-12, 6.6	0.541
Tp-Te V5 (ms)	79.04 (25.83)	79.47 (19.86)	-0.06	-8.1, 8.0	0.987
Tp-Te V6 (ms)	78.61 (28.70)	77.95 (17.05)	-1.3	-9.3, 6.8	0.759
Tp-Te/QT - V1	0.19 (0.06)	0.19 (0.04)	0.00	-0.02, 0.01	0.716
Tp-Te/QT - V2	0.20 (0.05)	0.20 (0.04)	-0.01	-0.02, 0.01	0.440
Tp-Te/QT - V3	0.20 (0.05)	0.19 (0.04)	-0.01	-0.03, 0.00	0.066
Tp-Te/QT - V4	0.19 (0.06)	0.19 (0.04)	-0.01	-0.02, 0.01	0.521
Tp-Te/QT - V5	0.18 (0.04)	0.19 (0.04)	0.00	-0.01, 0.02	0.555
Tp-Te/QT - V6	0.18 (0.05)	0.18 (0.04)	0.00	-0.01, 0.02	0.774
Sokolov (mm)	19.92 (7.46)	15.41 (5.24)	-4.4	-6.6, -2.2	<0.001
Cornell (mm)	12.55 (7.16)	12.72 (5.53)	-0.07	-2.3, 2.1	0.953
Intrinsicoid Deflection (mm)	49.18 (8.22)	46.23 (9.75)	-3.2	-6.5, 0.10	0.057

* values expressed as mean ± standard deviation. ^†^ Age-adjusted p-value calculated by linear regression. HR: heart rate. AH: arterial hypertension; CI: confidence interval.

The patients were also divided into resistant and non-resistant hypertension groups (Table 9).

**Table 9:** – Electrocardiographic measurements in the groups with or without resistant hypertension, and the estimated age-adjusted difference between the groups

Variables*	Resistant N=62	Non-resistant N=68	Adjusted difference	95% CI	p-value^**†**^
Total SBP (mmHg)	128.7 (14.0)	118.3 (10,3)	11.1	6.8, 15.3	<0.001
Total DBP (mmHg)	77.0 (10.3)	71.7 (8.8)	4.7	1.4, 8.0	0.006
Nº of Antihypertensives	4.76 (1.28)	2.54 (1.04)	2.2	1.8, 2.6	<0.001
HR (bpm)	63.97 (12.74)	63.76 (11.11)	-0.28	-4.5, 3.9	0.896
QT-V5 (ms)	433.30 (50.53)	420.96 (42.58)	16	0.33, 32	0.046
QTc-V5 (ms)	443.91 (49.32)	430.00 (37.35)	13	-2.1, 29	0.091
QTd	52.94 (28.20)	51.26 (28.63)	3.6	-6.2, 13	0.469
Tp-Te V1 (ms)	81.09 (28.55)	74.89 (20.15)	7.3	-1.5, 16	0.105
Tp-Te V2 (ms)	85.42 (26.95)	78.78 (16.99)	7.8	0.02, 16	0.049
Tp-Te V3 (ms)	84.65 (28.04)	78.73 (16.40)	6.9	-1.1, 15	0.090
Tp-Te V4 (ms)	84.98 (33.37)	77.15 (16.88)	8.5	-0.68, 18	0.069
Tp-Te V5 (ms)	84.46 (26.16)	74.68 (16.98)	11	3.1, 18	0.006
Tp-Te V6 (ms)	81.48 (27.41)	75.20 (16.01)	7.4	-0.49, 15	0.066
Tp-Te/QT - V1	0.19 (0.05)	0.18 (0.04)	0.01	-0.01, 0.03	0.188
Tp-Te/QT - V2	0.20 (0.05)	0.19 (0.04)	0.02	0.00, 0.03	0.050
Tp-Te/QT - V3	0.20 (0.05)	0.19 (0.04)	0.01	0.00, 0.03	0.069
Tp-Te/QT - V4	0.20 (0.06)	0.18 (0.04)	0.01	0.00, 0.03	0.090
Tp-Te/QT - V5	0.19 (0.05)	0.18 (0.04)	0.02	0.00, 0.03	0.019
Tp-Te/QT - V6	0.19 (0.05)	0.18 (0.03)	0.01	-0.01, 0.02	0.200
Sokolov (mm)	18.84 (6.81)	15.72 (6.01)	3.0	0.70, 5.2	0.011
Cornell (mm)	14.20 (7.18)	11.26 (4.80)	3.4	1.3, 5.5	0.002
Intrinsicoid Deflection (mm)	47.63 (9.54)	47.18 (9.06)	0.76	-2.5, 4.0	0.647

* values expressed as mean ± standard deviation; ^†^ Age-adjusted p-value calculated by linear regression; HR: heart rate. CI: confidence interval; SBP: systolic blood pressure; DBP: diastolic blood pressure.

To assess the intraobserver reproducibility of the measurements, the QT and Tp-Te intervals were selected to be retested on 10% of the total sample; that is, 13 ECGs were randomly selected and reanalyzed by the researcher after all study measurements were completed.

For the QT interval, the mean absolute error was 0.88 ms (SD ± 7.33 ms), representing a relative error of 1.46%, 95% CI -15.2 to +13.5 ms. For the Tp-Te interval measurement, the absolute error was 0.29 ms (SD ± 5.99 ms), representing a relative error of 5.91%, 95% CI -12.0 to +11.4 ms.

## Discussion

In our study, the patient profile consisted of hypertensive individuals with high cardiovascular risk. Compared to the study by Demir and Uyan,^12^ which served as the sample calculation basis for this study, patients with diabetes mellitus, chronic kidney disease (CKD), and obstructive sleep apnea were excluded. In that study, only 63% of patients were using combination therapy, as compared to 94% in our study.

These same exclusion criteria were applied by Karaagac et al.^13^. Additionally, the high cardiovascular risk profile and older age in our study, when compared to other studies, may explain the high prevalence of non-dippers (68%) in the studied population.

We found no significant differences between electrocardiographic measurements of ventricular repolarization and left ventricular hypertrophy (LVH) when comparing dipper and non-dipper groups.

Demir and Uyan^12^ studied 80 hypertensive patients (30 non-dippers), with an average age of 51.5 ± 8 in dippers and 50.6 ± 5.4 in non-dippers. Tp-Te intervals, Tp-Te/QT ratio, and QTd were significantly higher in non-dippers. They also showed an association with increased left ventricular mass indexes measured by echocardiography.

However, unlike our study, Demir and Uyan^12^ excluded patients with U waves and measured the endpoint of the T wave when the descending portion touched the isoelectric line without drawing a tangent. They also did not specify which leads represented their results.

Karaagac et al.^13^ evaluated 70 hypertensive patients with metabolic syndrome, with an average age of 55 ± 11 years (35 non-dippers). In lead DII, they found a significant increase in Tp-Te intervals (91 vs. 74 ms; p < 0.001), Tp-Te/QT (0.24 vs. 0.20 ms, p < 0.001), and Tp-Te/QTc (0.22 vs. 0.18 ms, p < 0.001) in non-dippers. However, this association was not observed for QT and QTc intervals or echocardiographic measurements^13^.

A larger study by Yan et al.^14^ selected 171 patients —102 elderly (>60 years) and 69 younger or middle-aged (<60 years) with treated AH. Only in patients <60 years was there an increase in QTc in non-dippers (438 ms vs. 416, p < 0.05), with no difference in Tp-Te. In the >60 group, no significant differences were found when comparing dipper, non-dipper, and reverse dipper groups^14^.

In a notable study, Passino et al.^15^ selected 48 untreated primary hypertensive patients, 20 of whom were classified as non-dippers, with a mean age of approximately 46 years and an average diagnosis duration of 1.2 years. This study evaluated electrocardiographic parameters over 24 hours, finding significantly higher QT and QTc intervals in non-dippers during sleep, while no significant difference was observed during the daytime period.

These findings highlight a lack of consensus in the literature and a lack of standardization in measuring the endpoint of the T wave across studies. It is also reasonable to consider that ventricular repolarization changes due to abnormal nocturnal dipping might be more evident during sleep rather than wakefulness.

Moreover, the differences in ventricular repolarization measures between dipper and non-dipper groups in our study may have been mitigated by the patients’ advanced age and higher comorbidity profile, as demonstrated by Yan et al.^14^

In our study, although we found a statistically significant increase in pro-BNP levels in the non-dipper profile, these values could not be associated with heart failure with reduced ejection fraction, as LVEF < 50% was an exclusion criterion for the study. Pro-BNP levels could be associated with a higher prevalence of heart failure with preserved ejection fraction. However, in the resting echocardiogram, this result also failed to reflect significant echocardiographic differences when comparing dipper and non-dipper groups.

This aligns with other literature findings, despite ongoing debate. For example, Cuspidi et al.^16^ studied 111 hypertensive patients with a recent diagnosis, with 33 non-dippers, and found no significant differences in LVH and carotid thickness between groups.

Roman et al.^17^ evaluated 183 hypertensive patients (79 non-dippers) and observed a higher prevalence of carotid plaques in non-dippers (41% vs. 27%). However, this significance was lost when adjusted for age, with no significant differences in left ventricular structure and systolic function between groups.

Ferrara et al.^18^ studied 179 hypertensive patients (123 long-standing hypertensives and 56 newly diagnosed) and found no difference in echocardiographic measures of diastolic dysfunction, left ventricular mass, or left atrial diameter between dippers and non-dippers.

A meta-analysis of 19 studies also found no significant correlation between nocturnal blood pressure dipping and LVH.^19^

By contrast, in a Chinese single-center study, a group of 183 hypertensive patients, whether on treatment or not, with an average age of 46.5 years and a hypertension duration of 5 years, included 117 non-dippers. This study demonstrated a significant increase in ventricular remodeling in non-dipper patients. These results were shown by an increase in LVi (101.3g/m^2^ in the non-dipper group, compared to 92.3g/m^2^ in the dipper group, p=0.029), associated with higher LV mass index rates, resulting in abnormal left ventricular geometry in 73.5% of the non-dipper group compared to 51.5% of the dipper group (p=0.001).^20^

Even more divergent results were obtained by Rodrigues et al.^21^ in a magnetic resonance study that evaluated 99 hypertensive patients, including 35 non-dippers. In this study, the non-dipper group was not associated with an increase in ventricular mass when compared to the dipper group; however, those classified as extreme dippers (nighttime SBP drop >20%) showed a significant increase in indexed ventricular mass and concentric ventricular hypertrophy^21^.

Although early studies published between 1988 and 1997 demonstrated an association between nocturnal BP decline and organ damage, such as carotid artery disease, LVH, and even stroke,^22-26^ larger and more contemporary studies have failed to reproduce these results. This has raised questions as to whether the non-dipper pattern as a marker of poor prognosis for cardiovascular events is related to nighttime hypertension or to other effects, including autonomic dysfunction, higher prevalence of CKD, diabetes mellitus, obstructive sleep apnea-hypopnea syndrome, and secondary hypertension.

The higher prevalence of methyldopa use in the dipper patient group can be explained by its pharmacological effect as an alpha-adrenergic blocker, considering that adrenergic stimulation is a very important mechanism for the abnormal nighttime decline in blood pressure, especially in patients with SAHOS. The use of alpha-adrenergic blockers has been associated with improved nighttime blood pressure, when compared to thiazide diuretics.^27^

The secondary analysis in our study showed that, when classifying patients based on AH resistance, the resistant group had significant increases in QT, Tp-Te, and Tp-Te/QT intervals, as well as higher Sokolow-Lyon and Cornell index values (Central illustration). These results do not appear to be associated with differences in SBP and DBP, as similar findings were not observed between controlled and uncontrolled AH groups.

This is the first published study demonstrating an association between resistant AH and ventricular repolarization changes, a clinically relevant finding. Salles et al. (2009) in a prospective cohort study with 538 patients with resistant AH, found that prolonged QTc was associated with increased risk of cardiovascular and all-cause mortality over a median follow-up of 4.8 years.^28^

1. The limitations of our study include:
2. The unicentric nature;
3. The electrocardiographic analysis being performed only during the wakefulness period;
4. The proportion of dipper and non-dipper patients, which may contribute to reducing the power of the results.
5. The lack of 24-hour or alternate-day ABPM for better classification of the groups.^29,30^
6. In our study, the fundamental criterion for inclusion was the recent performance of an ABPM examination, which may introduce selection bias, considering that the criteria used for indicating ABPM in routine practice include patients suspected of white coat effect, masked hypertension, resistant hypertension, patients with symptoms suggestive of hypotension, among others.
7. The measurement of ventricular repolarization performed solely by the tangent method, unlike other studies that used the tail method, although current literature suggests the tangent method as the most appropriate.

Finally, we hope that the data found in this study can assist and contribute to the literature so that we can understand the real association between nighttime blood pressure decrease and measures of ventricular repolarization.

## Conclusion

This was the first study conducted with the aim of analyzing ventricular repolarization in hypertensive dipper and non-dipper patients with this age profile and comorbidities, indicating a high cardiovascular risk profile, with a high prevalence of non-dippers.

In this population, we found no significant differences in clinical characteristics, demographics, and echocardiographic parameters when comparing dipper and non-dipper patients.

Regarding the electrocardiographic parameters of ventricular repolarization and left ventricular hypertrophy, when measuring the QT interval, QTc interval, Tp-Te, and Tp-Te/QT using the tangent method, no difference was found between dippers and non-dippers.

However, in patients with resistant hypertension, a significant increase was observed in the measures of ventricular repolarization. For this group of patients, the implementation of electrocardiograms in outpatient routine could be a useful tool in stratifying the risk of cardiovascular outcomes.
